# Long-term persistence of Chikungunya virus neutralizing antibodies in human populations of North Eastern Thailand

**DOI:** 10.1186/1743-422X-11-183

**Published:** 2014-10-21

**Authors:** Narong Nitatpattana, Kobkan Kanjanopas, Sutee Yoksan, Wichai Satimai, Narong Vongba, Sasiporn Langdatsuwan, Khajornpong Nakgoi, Supot Ratchakum, Nadia Wauquier, Marc Souris, Prasert Auewarakul, Jean-Paul Gonzalez

**Affiliations:** Center for Vaccine Development, Institute of Molecular Bioscience, Mahidol University, Nakhon Pathom, Thailand; Bureau of Vector Borne Disease, Department of Disease Control, Ministry of Public Health, Nontaburi, Thailand; Office Vector Borne Disease Control 6 Khon Kaen, Department of Disease Control, Ministry of Public Health, Nontaburi, Thailand; Sorbone Universités, UPMC, Univ Paris 06, CR7, CIMI-Paris, F-75005 Paris, France; Institut de Recherche pour le Développment, IRD, Vientiane, PDR Laos; Institute of Molecular Bioscience, Mahidol University, Nakhon Pathom, Thailand; Metabiota Inc, Sutter Street, suite 600, San Francisco, CA 94104 USA; Emerging diseases and Biosecurity, Metabiota, Inc, 8757 Georgia Avenue, suite 420, Silver Spring 20910, MD, USA & San Francisco, CA USA

**Keywords:** Chikungunya virus, Cross-neutralization, Thailand

## Abstract

**Background:**

Chikungunya virus (CHIKV) outbreak recurrences in Thailand are unpredictable and separated by unexplained and often long silent epidemiological periods that can last for several years. These silent periods could be explained in part by the fact that infection with one CHIKV strain confers lasting natural immunity, even against other CHIKV strains. In this study we evaluated the persistence of CHIKV-specific neutralizing antibodies in the population of Chumpae District, Khon Kaen Province, nineteen years after a CHIKV outbreak occurred in the same area in 1991.

**Findings:**

Overall 39% (44/111) of 111 former patients had neutralizing antibodies reacting against CHIKV ECSA strain. Consistently high titers of neutralizing antibodies were found in 75% (33/44) of all positively-reacting sera, 70% of which (23/33) were collected from individuals amongst the >60 years old age group. Although the prevalence found in Pong Haeng village (70%) was significantly higher than the prevalence detected in the Nong Thum village (14%), control study villages without known previous Chikungunya epidemics had a high Chikungunya neutralizing antibody prevalence (65%).

**Conclusions:**

More than one-third of the pre-exposed population had persisting natural immunity that was more likely boosted by recent and repetitive exposure to the emerging ECSA CHIKV in Thailand. Also, Chikungunya virus appears to largely circulate in the country with a great variability appears between villages or area probably associated with the vector abundance and efficiency. Altogether these results show a potential for a lifelong immunity against CHIKV. Given the rapid spread of the highly pathogenic ECSA strain in Southern Thailand, the development of CHIK vaccine is strongly recommended.

## Introduction

Since its discovery in 1952 in Tanzania [1], Chikungunya virus (CHIKV) has been responsible for numerous and recurrent outbreaks worldwide (see for review: [2]). The virus emerged in Southeast Asia in the late 1950s and in Thailand in 1958 [3]. CHIKV is an Alphavirus of the *Togaviridae* family clustering with the Old World alphaviruses and closely related to the African O’nyong’nyong virus. CHIKV is transmitted by mosquitos and hitherto has been responsible for chikungunya fever, a dengue-like illness in humans, characterized by fever, rash and characteristic severe and persistent arthralgia. These late and major clinical symptoms affect the small joints in particular and are often associated with excruciating pain [4]. The disease is generally non-fatal and the acute phase resolves within 3 to 4 days whereas the arthralgia symptoms may persist for sometimes weeks or months.

Recurring epidemics are observed when CHIKV accidentally spills over from its sylvatic transmission cycle to the human population. The natural cycle of CHIKV involves several amplifying mammal hosts including primates, sheep, rodents, bats, as well as birds and forest-dwelling *Aedes spp*. mosquito vectors [5]. In Thailand, outbreak recurrences have been unpredictable with silent inter-epidemic phases that can last for more than a decade [6, 7].

However, in 2005, a new East Central and South African (ECSA) CHIKV strain emerged in the Indian Ocean, changing the epidemiological pattern of the disease with an increase of infectiousness, morbidity, severity and efficiency of transmission from the vector [8]. Indeed, the newly acquired A226V glycoprotein E1 mutation of ECSA strain conferred, among other potential properties, an advantage in vector competence (i.e.: infectivity) of a well-distributed mosquito in Asia, *Ae. Albopictus* [8–10]. This mutation appeared independently during several recent outbreaks in different locations where *Ae. albopictus* is predominant including La Reunion, Cameroon, Gabon and Thailand [7, 11, 12] and ECSA was also responsible for several major outbreaks in Southeast Asia that mostly struck southern Thailand [9]. Besides the potential of such a mutation on infectivity and transmission effectiveness, the severity of the outbreaks could also be accentuated by the lack of pre-existing antibodies in the population [13]. Indeed CHIKV in Asia has been responsible for sporadic and sometimes explosive urban outbreaks amongst non-immune populations in the last two decades [14, 15]. Moreover neutralizing antibodies (nAb) to CHIKV are generated during natural infection in humans and several sero-surveys as well as experimental studies have suggested that nAb prevent virus replication conferring a potentially important protective role for nAb in the development of secondary CHIKV infections [16–18].

In the present study we targeted a population that had been primarily exposed and infected by the Asian CHIKV genotype in 1991 and showed the persistence of high levels of potentially protective neutralizing antibodies against several CHIKV genotypes in the same individuals, almost two decades after primary infection.

## Material and methods

### Study population and area

**Figure 1:**
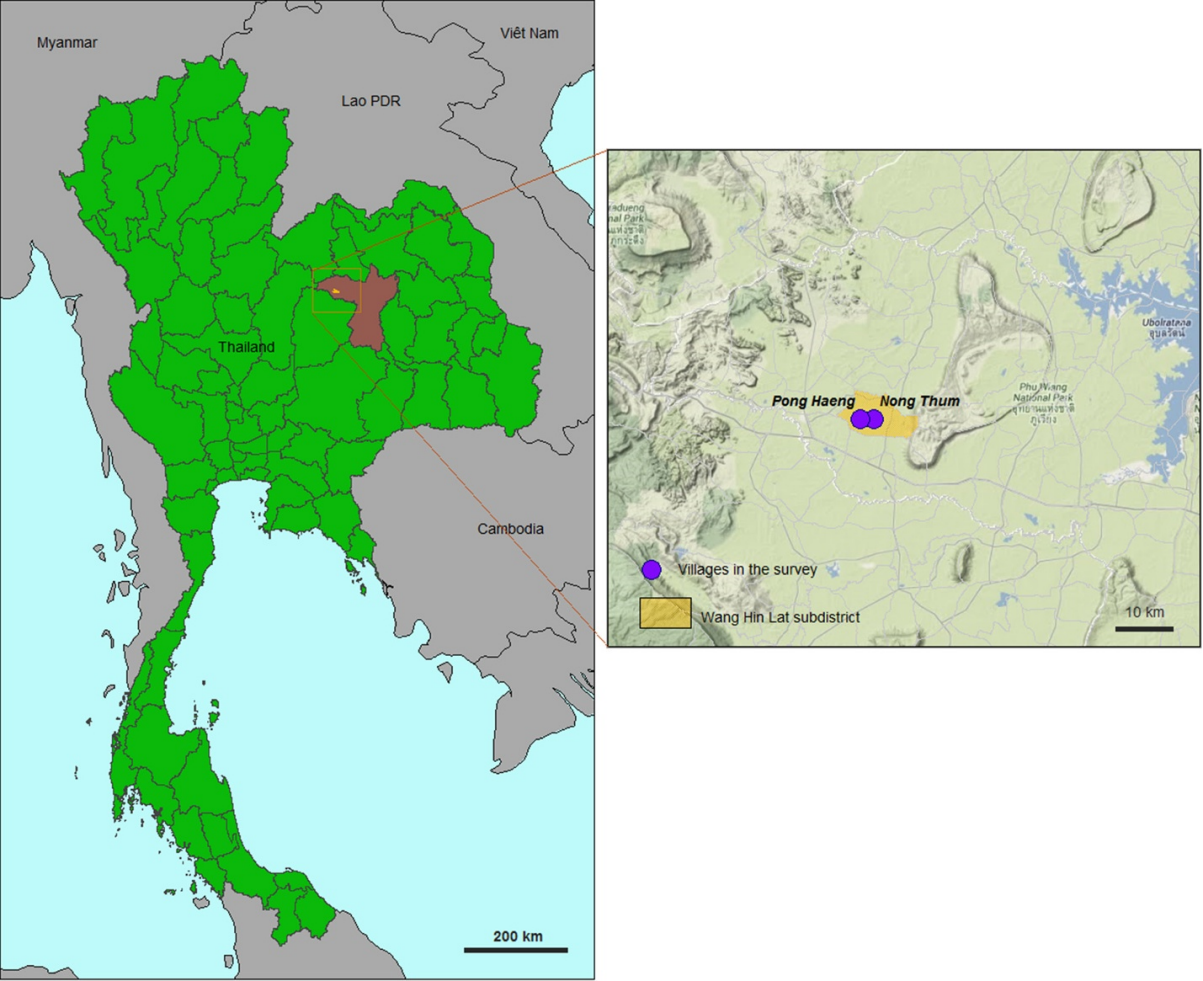
**Study sites.** Legend: Left: Kingdom of Thailand (green); Khon Kaen Province (brawn); Wang Hin Lat sub district (yellow). Right: Wang Hin Lat sub district (yellow); The two study villages (=Thai: *ban*) of Pong Haeng (16°36′45″N, 102°3′35″E) and Nong Thum (16°36′47″N, 102°4′50″E) distant for less than 1.5 to 2 km one from the other.

In 1991 an outbreak of Asian CHIKV genotype hit the Province of Khon Kaen, causing 262 cases in the villages of Pong Haeng (16°36′45″N, 102°3′35″E) and Nong Thum (16°36′47″N, 102°4′50″E), Wang Hin Lat sub district, Chumpae district, Khon Kaen Province, northeastern region of Thailand of Thailand (Figure 1) [6]. In 2010, 111 individuals out of the 262 original cases were sought out and sampled in order to further study the herd immunity against CHIKV. Oral consent was obtained under the guidance of the Ministry of Health and the Mahidol University Ethical Committee (# MU_IRB 20101/325.2511). Blood was drawn (3 mL) from 19 years old and older volunteers who have lived in the study area since the outbreak.

A control population was tested for Chikungunya neutralizing antibodies from three nearby villages of the study site including Nong Pinia (16° 36′ 62″N 102° 4′ 89″E), Nade (16° 36′ 58″N 102° 4′ 59″E) and, Sok Udom (16° 36′ 60″N 102° 4′ 74″E) previously free of Chikungunya reported cases during the 1991 epidemic of the Khon-Khen Province.

### Plaque Reduction Neutralization Test (PRNT)

Virus–antibody interaction was performed in test tube, and antibody effects on viral infectivity recorded by incubating the virus-antibody mix on plated susceptible Vero cells as described in [19, 20]. Vero cells were used for both virus production and PRNT. The CHIKV strain used in the assay belongs to the ECSA CHIKV genotype isolated in Thailand in 2008 (08/364AF strain) (Yoksan, personal comm.). Briefly, as previously described [21] and modified [22] cells were seeded in 6-well plates at 1 × 10^5^ cells/well, incubated for 5–7 days to reach confluence. Sera samples were diluted 1:5, followed by 10-fold serial dilutions in PBS pH 7.5 with 30% fetal bovine serum (FBS), mixed with virus (starting dilution 1:10) and incubated at 37°C for 7 days. Ninety minute after addition of the mix virus/serum, cells were overlaid with 3.0% carboxymethyl cellulose with added neutral red and 50 visualized plaques were then counted. Results were interpreted using the Probit model with the SPSS® program, and PRNT end point titers were expressed as the reciprocal value of the last serum dilution. PRNT titer was defined as the highest serum dilution inducing a 50% reduction (PRNT50) in plaque counts [20]. Samples with PRNT titers lower than 1:10 were considered as negative. Positive and negative controls were also added including patient sera tested positive by IFA (IgG/IgM of Chikungunya Antibodies with “Reflex(es) to Titer” from Focus Diagnostics®) and, negative controls using normal human serum tested negative by IFA cells density as above.

## Results and discussion

In 1991, during the CHIKV outbreaks of the Chumpae sub district, the Ministry of Health tested 262 sera collected from CHIKV-infected patients and showed a 4-fold rise of hemagglutination inhibition in 82.5% of cases [6, 23]. Nineteen years later, we collected 111 sera samples from the same group of former patients and tested them for the presence of neutralizing antibodies using PRNT. Overall 39% (44/111) of former patients had neutralizing antibodies against the CHIKV ECSA strain (Table 1). Consistently high titers of neutralizing antibodies >10^3^ PRNT50 were found in 75% (33/44) of all positively-reacting sera, 70% of which (23/33) were collected from individuals amongst the >60 years old age group. Moreover the prevalence found in Pong Haeng village (70%) was significantly higher (p < 0.001) than the prevalence detected in the Nong Thum village (14%) (Table 2).

**Table 1:** **Chikungunya virus (ECSA strain) neutralizing antibody prevalence among the population of Pong Haeng and Nong Thum villages (Khon Khen Province, Thailand) tested in 2010 and pre-exposed to the Chikungunya outbreak of 1991**

Age	PRNT50 titer		Positive/Tested (%)
	10 - 10 ^3^	>10 ^3^	
19 – 34	1	1	2/14 (14.3)
35 – 50	2	9	11/37 (29.7)*
>50	8	23	31/60 (51.7)#
Total (%)	11 (9.9)	33 (29.7)	44/111 (39.6)

Chi square test by age classes: *19/34 to 35/50, chi-square: 6.42, p = 0.011; #19/34 to >50, chi-square = 1.27, no significant.

**Table 2:** **Comparative serology of CHIK (ECSA strain) neutralizing antibodies among the population of the two study villages, Khon Kaen 2010**

Village	None immune	Immune (%)	Total
Pong Haeng	16	37 (69.8)*	53
Nong Thum	50	7 (12.1)	58
Total	66	44 (39.6)	111

*Chi square *p < 0.001*.

Although one can estimate that the herd immunity was globally reduced by half (82.5% positive in 1991 against 39% positive in 2010) after nineteen years, more than one third of the population previously exposed to the CHIKV epidemic in Chumpae district still presented a consistent level of neutralizing antibodies. Importantly, this immunity, primarily triggered in 1991 by the Asian CHIKV genotype, showed potential cross-reactive activity with the newly imported ECSA CHIKV in Asia [24]. Although, no CHIKV epidemics were reported from Khon Kaen Province during nineteen years, major outbreaks caused by the ECSA strain occurred in southern Thailand from 2008 to 2010 and the virus was thought to actively circulate throughout the country [9]. Altogether, it is reasonable to hypothesize that the observed high titers of neutralizing antibodies could be attributed to multiple exposures to the ECSA strain imported to the Khon Kaen Province that ultimately boosted the natural protection acquired by the inhabitants of Chumpae. From July 17 to September 13, 1991, the village of Pong Haeng (454 inhabitants) reported 262 CHIK cases (58% of the population), while later on, from September 10 to 14, Nong Thum village (874 inhabitants) reported only 69 patients (8%). The spread of the infection to Nong Thum was considered to be a spillover from the first village (reported by: Kitisriworapoj SJ, Division of Epidemiology, Ministry of Public Health, Kingdom of Thailand). Moreover, in August 1991, following the onset of the Pong Haeng outbreak, a large mosquito-control campaign was launched in the district in order to decrease virus transmission, and ultimately reduce the disease incidence in secondary sites that were not yet affected at that time such as Nong Thum village. These events easily explain the low incidence of CHIKV cases in Nong Thum village and the lower prevalence observed two decades later.

However, surprisingly, when we compare the present results to the nearby control villages that never reported CHIK fever cases, at least during the two past decades, the global seroprevalence appears two times higher (65%) as for the study villages (not significant p > 0.1) (Tables 3 and 4). Also, from the literature, we have to consider that the population control was repeatedly exposed to the less pathogenic CHIKV strain that is largely producing asymptomatic infection [25, 26].

**Table 3:** **Chikungunya virus (ECSA strain) neutralizing antibody by age classes among the population of villages (see Table** 4 **) of the Khon-Khen Province (2010) where no clinical cases of Chikungunya were reported to the MOH during the 1991 Chikungunya**

Age	PRNT 50 titer		Positive/Tested
	10 - 10 ^3^	>10 ^3^	
19 - 34	0	0	0/3
35- 50	3	15	18/27
>50	2	6	8/10

**Table 4:** **Chikungunya virus (ECSA strain) neutralizing antibody among the population of three villages of the Khon-Khen Province (2010) where no clinical cases of Chikungunya were reported to the MOH during the 1991 Chikungunya outbreak in the Province**

Village	Positive/Tested
Nong Pinia	11/20
Nadee	4/6
Sok Udom	11/14
Total	26/40 (65.0%)

Caption. Location of non infected villages during the 1991 Chikungunya outbreak in the Khon-Khen Province: Nong Pinia (16° 36′ 62″N; 102° 4′ 89″E); Nadee (16° 36′ 58″N; 102° 4′ 59″E); Sok Udom (16° 36′ 60″N; 102° 4′ 74″E).

From the control population, despite the limited number tested, positive samples are entirely find in the older (>34 yo) population with a predominantly high titer (5/21) suggesting multiple exposure before the national policy for Dengue mosquito control [27].

Naturally acquired immunity has been shown to be crucial during CHIKV infection and different strains generate effective cross protection against each other [13]. Eventually the high neutralizing antibody titer observed in the elderly inhabitants, appears more likely due to sequential subclinical infections that occurred during the CHICKV inter-epidemic silent phase in the Hin Lat sub district, definitely associated to the ECSA CHIKV strain newly imported in Thailand and efficiently transmitted by *Ae. albopictus,* widely distributed across the country [7].

The geographical spread of ECSA CHIKV strain will certainly change the epidemiological pattern of CHIKV in Thailand. Given the pattern of emergence, spread and occurrence of serial outbreaks of the ECSA CHIKV genotype in Asia, it is likely that ECSA is already well installed in a natural cycle involving the abundant and highly competent *Ae. albopictus* on one hand and the important population of primate macaques known to be naturally infected and thought to be the natural host reservoir of CHIKV in Thailand on the other hand [22, 28]. Ultimately naturally pre-exposed population to CHIKV develop, if not a lifelong, a durable immunity that can be eventually be boosted by subclinical infections with any circulating strain of CHIKV [24, 29]. With the emergence of the ECSA CHIKV genotype in Asia, the invasiveness of *Aedes albopictus* as a major vector of CHIKV, the epidemiological pattern of virus transmission will certainly evolve targeting the largely non-immune dense population of the urban area of Southeast Asia. Ultimately, the potential for a naturally acquired CHIKV long term immunity play in favor favor of the development of CHIKV vaccine in order to achieve in a short term an efficient herd immunity that will control future epidemics.
